# Enhancing the Appeal of Clinical Diagnostics Education for Medical Undergraduates Through the Innovative Incorporation of Cultural and Historical Elements Alongside the Inclusion of the COVID-19 Pandemic and Its Innovative Solutions

**DOI:** 10.7759/cureus.66261

**Published:** 2024-08-06

**Authors:** Ning Zhang, Yuanyuan Zhu, Lin Kang, Xiaoming Huang

**Affiliations:** 1 Department of Geriatrics, Peking Union Medical College Hospital, Peking Union Medical College, Chinese Academy of Medical Sciences, Beijing, CHN; 2 Department of Cardiology, Peking Union Medical College Hospital, Peking Union Medical College, Chinese Academy of Medical Sciences, Beijing, CHN; 3 Department of General Medicine, Peking Union Medical College Hospital, Peking Union Medical College, Chinese Academy of Medical Sciences, Beijing, CHN

**Keywords:** real cases, old historical photos, historical documents, traditional chinese cultural classics, clinical diagnostics

## Abstract

Clinical diagnostics is a fundamental course required for clinical medical students and serves as a prerequisite for several advanced clinical subjects. However, recent observations indicate a decline in interest among eight-year clinical medicine students at Peking Union Medical College regarding clinical diagnostics courses. Instead, these students seem to prioritize the publication of high-impact articles and involvement in scientific research over their medical coursework, leading to a lack of sufficient attention to clinical diagnostics.

In the clinical diagnostics course conducted in the first half of 2024, our objective was to engage medical students by presenting the subject matter in an interesting and relevant manner. We curated textual information regarding the health condition of Lin Daiyu, the protagonist from the Chinese literary classic "The Dream of the Red Chamber," and encouraged students to deduce potential diseases she may have experienced based on the original text. Additionally, we sourced historical photographs of Empress Dowager Cixi from the Qing Dynasty, which facilitated the hypothesis that she likely suffered from goiter. These images were employed as a practical examination question during the mid-semester assessment to evaluate the students' proficiency in conducting neck physical examinations. Furthermore, we shared an inspiring anecdote about healthcare professionals who repurposed potato chip packaging into stethoscopes during the COVID-19 pandemic, underscoring the critical role of physical diagnosis and examination in urgent situations. Following the mid-term exam in clinical diagnosis, a questionnaire survey was administered to the medical students who participated in the examination. The results indicated that 93% of the students found the question regarding Lin Daiyu to be highly engaging, while 89% found the question about Empress Dowager Cixi equally captivating. These innovative teaching strategies significantly enhanced the medical students' enthusiasm for learning clinical diagnostics.

## Editorial

Clinical diagnostics is a critical discipline in the medical field that focuses on theories, knowledge, skills, and diagnostic reasoning essential for identifying diseases. It serves as a foundational course following basic medical studies in areas such as human anatomy, physiology, medical microbiology, and pathology. This discipline explores a wide range of clinical manifestations and disease mechanisms, including consultation and physical examination techniques, laboratory and auxiliary examinations, and the application of scientific thinking for accurate disease diagnosis. Proficiency in clinical diagnostics enables students to effectively communicate with patients, establish strong doctor-patient relationships, and develop the necessary skills to collect, analyze, and synthesize information for precise disease diagnosis. This knowledge not only aids in disease prevention and treatment but also helps students evaluate prognosis and plan for rehabilitation. Ultimately, clinical diagnostics serves as a gateway to various clinical disciplines, acting as a vital link that enhances the practice of clinical medicine [1].

The “Clinical Diagnostics” course for eight-year clinical medicine students at Peking Union Medical College is divided into two semesters, starting in the fourth year of their studies. The first semester focuses on theoretical classes related to physical examination, while the second semester covers symptomology, consultation, medical record writing, and auxiliary examinations. In the physical examination courses, six to eight senior attending physicians from various specialties at the Department of Internal Medicine in Peking Union Medical College Hospital are chosen to lead team teaching for the annual clinical diagnostics course. These physicians instruct specific physical examination methods. Each teaching group typically consists of one teacher and five to seven medical students, who participate in physical examination exercises for three hours every Wednesday and Friday afternoon in a designated classroom. The students collectively review pre-recorded videos demonstrating standard physical examination procedures and then engage in one-on-one practice sessions. The teacher evaluates the students' technique for correctness and standardization, identifies any missed examination components, and addresses student questions after the exercise. In situations where patients show typical symptoms and signs in various hospital wards, with the patient's and family's consent, the teaching teacher accompanies the students to the ward to observe the patient's symptoms and signs at the bedside. This hands-on experience is designed to enhance the students' understanding of typical symptoms and signs.

This teaching model has been established for more than four decades. In recent times, there has been a decrease in interest in conventional theoretical lecture styles among a subset of medical students. Within the classroom, specific students have been noticed not actively participating in the professor's presentation, choosing instead to utilize their laptops for activities such as viewing slides, reading literature, writing papers, or even playing games. When questioned about why a number of their classmates do not focus during lectures, some medical students mentioned that they perceive the material as uninteresting and favor self-directed learning through textbooks and slides.

Fourth-year medical students experience substantial pressure to engage in scientific research alongside their theoretical coursework. During this period, students typically select research mentors and immerse themselves in various projects, spanning from basic research with animal experiments or molecular biology tests to clinical research involving data collection from multiple patients, clinical follow-ups, and statistical analysis. Hospitals frequently prioritize the publication of impactful articles when evaluating job candidates, prompting medical students to prioritize publishing articles over participating in clinical internships and courses.

Given our observation that medical students tend to prioritize scientific research over clinical courses, we aim to implement innovative changes to the traditional teaching methods of clinical diagnostics. Our goal is to enhance engagement and foster greater interest in this essential course among medical students. Our idea was inspired by traditional Chinese cultural classics, historical documents, and old historical photos, with a significant influence from “The Dream of the Red Chamber,” one of the four great classics of ancient China. Authored by Cao Xueqin during the Qing Dynasty, the novel consists of 120 chapters, with the first 80 chapters credited to him and the remaining 40 chapters written by an anonymous author, later edited by Cheng Weiyuan and Gao E. “The Dream of the Red Chamber” revolves around the fortunes and misfortunes of the four aristocratic families - Jia, Shi, Wang, and Xue - centered on the wealthy young master Jia Baoyu. The narrative primarily explores the love and marriage tragedies involving Jia Baoyu, Lin Daiyu, and Xue Baochai while also delving into the lives of various boudoir beauties. Through its depiction of humanity and tragedy, the novel offers a profound insight into the beauty of women and the multifaceted nature of ancient Chinese society [2]. Lin Daiyu, the heroine in “The Dream of the Red Chamber,” is a classic female character deeply rooted in ancient literary works. She was known for her intelligence and beauty, traits that were evident from a young age and greatly valued by her parents. Lin Daiyu's educational journey began at the tender age of five, but tragedy struck early in her life with the loss of her mother when she was only six to seven years old, followed by her father's passing when she was 11. Raised and educated by her mother's family, the Jia family, Lin Daiyu grew to possess a solitary and proud demeanor. At the age of 12, she took up residence in the Xiaoxiang Pavilion within the Grand View Garden, a dwelling originally built for Concubine Yuan, where she was reunited with Jia Baoyu, her childhood sweetheart. “The Dream of the Red Chamber” beautifully recounts the poignant tale of Lin Daiyu's unrequited love for Jia Baoyu [3]. Lin Daiyu is a celebrated figure in Chinese literature, and excerpts from “The Dream of the Red Chamber” are often included in high school Chinese language textbooks. For quite some time, literary enthusiasts speculated that Lin Daiyu meets a tragic end due to tuberculosis in “The Dream of the Red Chamber.”

The description of Lin Daiyu's condition in “The Dream of the Red Chamber” may seem disjointed; however, when examined through a modern diagnostic lens, discernible clues emerge. We posed a question to medical students studying for clinical diagnostics exams centered around Lin Daiyu's condition in the novel as follows: “As a diligent student, you found yourself transported into the world of the Chinese classic 'The Dream of the Red Chamber' after falling asleep while studying for the mid-semester exam. Within this new world, you encountered Lin Daiyu's case summary, describing her as a young individual who has been physically underdeveloped since childhood. Despite her age, she displays good behavior and communication skills, along with a timid yet naturally charming demeanor that suggests potential underlying health issues. Lin Daiyu experiences chronic cough and difficulty breathing following the spring and autumn equinoxes, with advanced stages marked by the presence of blood in her sputum. Her breathing difficulties worsen after consuming more water, as doctors have advised her to limit her water intake due to her condition. Symptoms escalate at night, as seen in a specific incident where she suffered from hoarse coughing, facial redness, swelling, and breathlessness, prompting her maid Zijuan to intervene. Given these symptoms, you are considering performing a heart auscultation on Lin Daiyu. Your inquiries are as follows: What components should be included in the auscultation? What abnormal findings might be detected during the cardiac auscultation of Lin Daiyu?”

The first question evaluates medical students' proficiency in cardiac auscultation. The second question, which is open-ended, focuses on the character Lin Daiyu, created by a novelist, leading to varied interpretations among students. Some students hypothesized that Lin Daiyu suffered from mitral valve stenosis, while others suggested chronic heart failure. The primary aim of the second question is to assess students' reasoning skills following diagnostics training, with points awarded for well-founded and logically rigorous reasoning. By utilizing the study of classics, this exercise challenges students to apply professional knowledge in “solving cases,” providing valuable insights for future clinical diagnoses.

**Figure 1:**
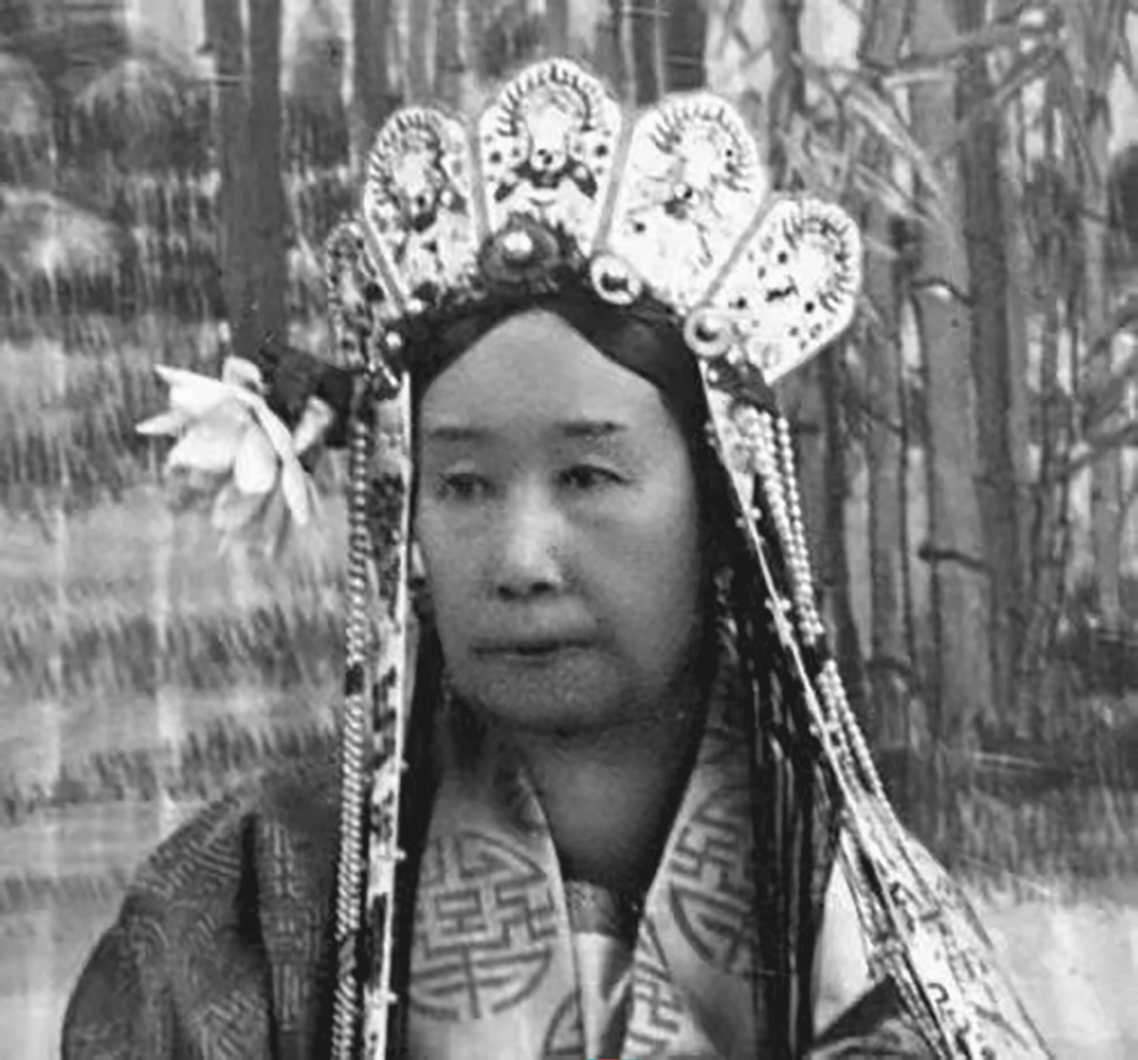
Photo of Empress Dowager Cixi in her old age. Image source: Yang S, Xu X. Goiter in the Qing Dynasty. Hormones (Athens). 2024;23 (2):267-268. This image is officially authorized.

Meanwhile, in the mid-term examination of Clinical Diagnostics, medical students were presented with a question based on a historical document concerning Empress Dowager Cixi of the Qing Dynasty in China. As the de facto ruler of imperial China's Qing government from 1861 to 1908, Empress Dowager Cixi occupies a significant position in modern Chinese history. During the period when photography was introduced to China, coinciding with the Qing government's isolationist policies, only members of the royal family and nobility within the palace had access to photography. Empress Dowager Cixi was one of the most frequently photographed individuals and left behind a substantial collection of images. In a photograph taken in 1903, the 68-year-old Cixi is depicted with a visibly swollen neck, indicative of a goiter (Figure 1). The symptoms of goiter were first documented in a Chinese herbal treatise titled “Pen-Tsao Tsing” around 3000 BC, while its association with exophthalmos was first noted by a Persian physician in the 12th century. Based on the available evidence, it is probable that Empress Dowager Cixi suffered from a simple goiter. These findings suggest that thyroid disorders were not uncommon during the Qing Dynasty despite the limited documentation regarding the prevalence and treatment of such conditions [4].

Medical students were presented with a short answer question based on historical documents from the Qing Dynasty. The question instructed them to examine the photograph of Empress Dowager Cixi in her later years, with particular emphasis on her neck (Figure 1). Specifically, they were asked to consider what they might observe if they were present at the time the photograph was taken and conducted a physical examination of her neck. The primary objective of this section is to evaluate the students' understanding of neck examination techniques.

Following the clinical diagnosis mid-term exam, a questionnaire survey was administered to 80 medical students who participated in the examination. The results indicated that 93% of the students found the question regarding Lin Daiyu to be highly engaging, while 89% found the question about Empress Dowager Cixi equally captivating. Overall, the students demonstrated a significant interest in the questions presented.

**Figure 2:**
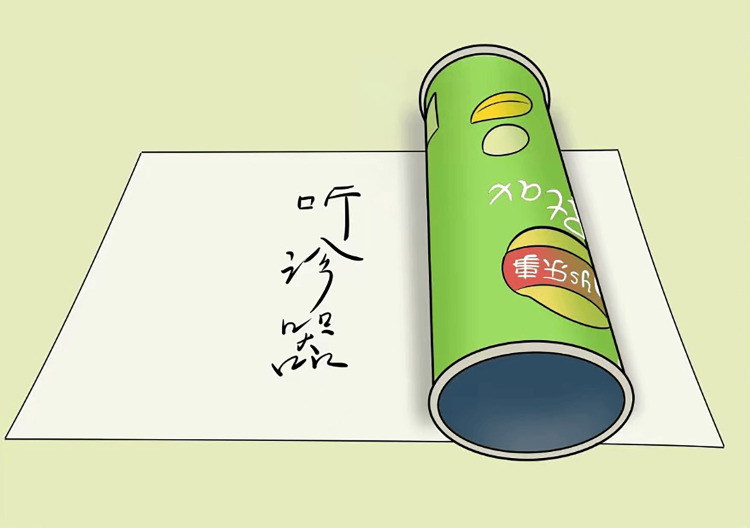
An empty potato chip cylinder, measuring 20-30 cm in length and 5-8 cm in diameter, was wrapped with a piece of sterile A4 paper to create a makeshift stethoscope. This cartoon was drawn by Dr. Yuanyuan Zhu.

**Figure 3:**
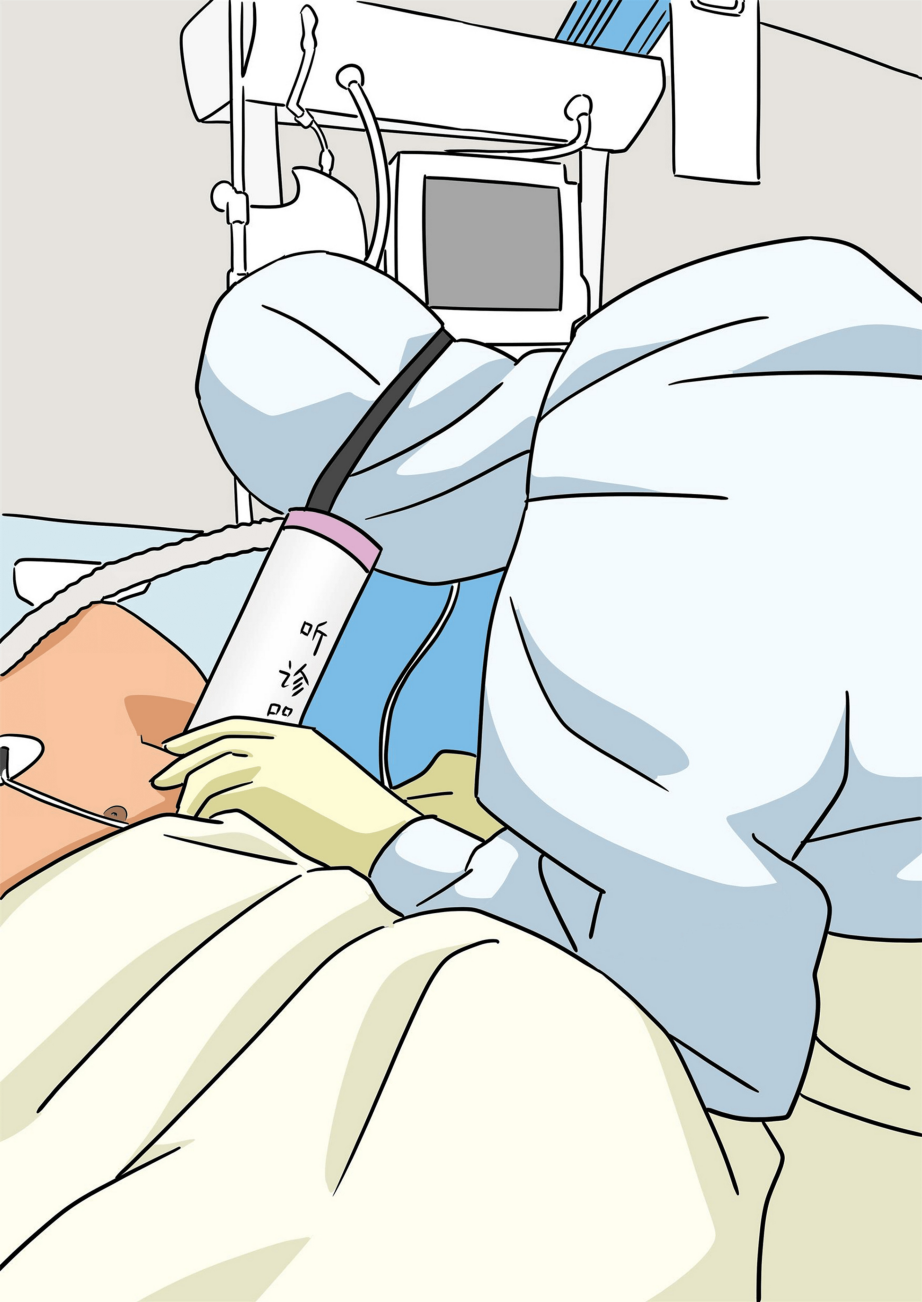
During the COVID-19 pandemic, a Chinese doctor creatively repurposed long potato chip tubes into stethoscopes to auscultate the lungs of patients suffering from severe COVID-19 infections. This cartoon was drawn by Dr. Yuanyuan Zhu.

With the rapid advancement of medical technology, a diverse array of advanced diagnostic tools has become available. Nonetheless, it is essential to underscore the continuing significance of physical examination and clinical diagnostics. An intriguing case was presented during clinical teaching sessions for medical students. When the COVID-19 epidemic emerged, frontline doctors required specialized tools to assess patients' conditions during diagnosis and treatment. In outpatient settings, physicians employed diagnostic techniques, such as visual inspection, palpation, percussion, and auscultation, to preliminarily detect lung infections, utilizing a stethoscope. In inpatient wards, where patients' conditions could change rapidly, immediate responses and root cause identification were critical, often necessitating the use of a stethoscope. However, conventional in-ear stethoscopes proved impractical due to the thick protective suits worn by doctors. Even when a stethoscope was pre-inserted into the suit, the risk of cross-infection remained high when used on multiple patients. Dr. Tan Yan, who was involved in treating severe COVID-19 cases, drew inspiration from an image depicting an early physician using a wooden tube for auscultation in the absence of a stethoscope. This led him to innovate and create his own stethoscope using locally available materials. Initially considering a badminton tube due to his recreational interest, he found it too lengthy for effective auscultation and instead repurposed a long potato chip tube (see Figure 2 and Figure 3). The resulting homemade stethoscope proved simple to construct, demonstrated significant efficacy, and minimized the risk of cross-infection. Over 300 patients benefited from this locally crafted stethoscope during the COVID-19 crisis, garnering praise and adoption at medical facilities, such as Wuhan Huoshenshan Hospital, where it was highly esteemed by frontline healthcare workers [5].

When explaining this case to medical students, it is essential to underscore the importance of anchoring their understanding in clinical practice. Encouraging them to recognize challenges within a clinical setting, utilize creativity to devise solutions, and subsequently disseminate their findings to a broader audience is crucial.

The lack of interest among medical students in foundational medical knowledge presents a critical challenge. Despite recognizing the importance of this knowledge for their future careers, students often prioritize scientific research and article publication to enhance their employability upon graduation, a trend exacerbated by the current evaluation system that predominantly emphasizes scientific research output. However, clinical skills and basic medical knowledge are essential prerequisites for effective medical practice. It is concerning to envision that today's medical students - who will become tomorrow's physicians - may lack the fundamental skills and knowledge necessary to drive advancements in clinical practice. Acknowledging this dilemma, we strive to cultivate medical students' enthusiasm for learning by utilizing engaging, dynamic, and thought-provoking examples, which are currently scarce in the medical education landscape in China. By exploring clinical diagnostic teaching materials derived from ancient Chinese literary masterpieces, historical texts, vintage photographs, and real case studies, we aim to enhance the appeal of the clinical diagnostics course and provide students with a sense of fulfillment. This approach has the potential to ignite a profound interest in clinical medicine among students. We believe that our limited experiences can serve as a valuable resource and inspiration for fellow educators involved in teaching clinical diagnostics.
